# PIAS1 Shapes a Tumor-Suppressive Microenvironment by Suppressing Immune Evasion in Oral Squamous Cell Carcinoma

**DOI:** 10.3390/cancers17172905

**Published:** 2025-09-04

**Authors:** Parisa Ghahremanifard, Jinsu An, Ayan Chanda, Angela M. Y. Chan, Steven C. Nakoneshny, T. Wayne Matthews, Shamir P. Chandarana, Robert D. Hart, Martin D. Hyrcza, Joseph C. Dort, Shirin Bonni, Pinaki Bose

**Affiliations:** 1 Department of Biochemistry and Molecular Biology, Cumming School of Medicine, University of Calgary, Calgary, AB T2N 4N1, Canada; parisa.ghahremanifar@ucalgary.ca (P.G.); jinsu.an@ucalgary.ca (J.A.); achanda@ucalgary.ca (A.C.); sbonni@ucalgary.ca (S.B.); 2 Charbonneau Cancer Institute, Cumming School of Medicine, University of Calgary, Calgary, AB T2N 4N1, Canada; angela.chan3@albertahealthservices.ca (A.M.Y.C.); scnakone@ucalgary.ca (S.C.N.); wmatthew@ucalgary.ca (T.W.M.); martin.hyrcza@ucalgary.ca (M.D.H.); jdort@ucalgary.ca (J.C.D.); 3 Cancer Translational Research Core, Arthur JE Child Comprehensive Cancer Centre, Alberta Health Services, Calgary, AB T2N 4N1, Canada; 4 Ohlson Research Initiative, Arnie Charbonneau Cancer Institute, Cumming School of Medicine, University of Calgary, Calgary, AB T2N 4N1, Canada; shamir.chandarana@ucalgary.ca (S.P.C.); robert.hart@ahs.ca (R.D.H.); 5 Department of Surgery, Section of Otolaryngology—Head and Neck Surgery, Cumming School of Medicine, University of Calgary, Calgary, AB T2N 4N1, Canada; 6 Department of Pathology and Laboratory Medicine, Cumming School of Medicine, University of Calgary, Calgary, AB T2N 4N1, Canada; 7 Department of Oncology, Cumming School of Medicine, University of Calgary, Calgary, AB T2N 4N1, Canada

**Keywords:** oral squamous cell carcinoma, PIAS1, single-cell RNA-seq, tumor microenvironment

## Abstract

Background: The SUMO E3 ligase PIAS1 (Protein Inhibitor of Activated STAT1) regulates pathways such as TGFβ signaling and has been implicated in multiple cancers. However, its role in the tumor microenvironment (TME), particularly in non-malignant stromal and immune cells, remains poorly understood. This study aimed to characterize the expression and functional relevance of PIAS1 within the TME of oral squamous cell carcinoma (OSCC). Methods: PIAS1 protein expression was assessed via immunohistochemistry (IHC) on OSCC tissue microarrays. Single-cell RNA-sequencing (scRNA-seq) datasets from OSCC tumors and normal tissues were analyzed to map cell-type-specific PIAS1 expression. Downstream effects were evaluated using differential gene expression, Ingenuity Pathway Analysis (IPA), gene set enrichment analysis (GSEA), and cell–cell communication inference. Results: IHC analysis revealed that higher stromal PIAS1 levels correlated with improved survival. scRNA-seq analysis showed an increase in the proportion of PIAS1-expressing cells across most stromal and immune cell populations within OSCC-derived tumors compared to their counterparts in adjacent normal tissue. However, when comparing PIAS1-positive cells, expression levels were significantly reduced in cancer cells, CAFs, TAMs, T cells, and endothelial cells within the TME. PIAS1-positive CAFs, TAMs, and T cells exhibited activation of apoptotic and tumor-suppressive pathways, while PIAS1-negative counterparts showed enrichment of immunosuppressive signaling and immune checkpoint expression. Cell–cell communication analyses indicated that PIAS1 fosters an immune-activated TME by promoting pro-inflammatory signaling, M1-like TAM polarization, and T cell activation. Conclusions: PIAS1 expression in stromal and immune cells is associated with tumor-suppressive reprogramming of the OSCC microenvironment. These findings position PIAS1 as a potential modulator of anti-tumor immunity and candidate target for therapeutic intervention.

## Simple Summary

PIAS1 is a protein that helps control how cells behave, including how they respond to signals that can lead to cancer. While it has been studied in cancer cells before, we wanted to know what it does in the cells around the tumor, the tumor microenvironment, where immune and other supporting cells live. We found that higher levels of PIAS1 in these surrounding cells were linked to better survival in patients with oral cancer. By studying individual cells from tumors, we saw that PIAS1 was often found in immune and support cells and seemed to help these cells fight cancer. When PIAS1 was missing, the cells showed signs of being more friendly to the tumor and less active in fighting it. Overall, PIAS1 appears to play an important role in helping the body’s own cells resist cancer and could be a useful target for future treatments.

## 1. Introduction

Oral squamous cell carcinoma (OSCC) is the most common head and neck cancer (HNC) and is associated with high morbidity and mortality. Despite significant advances in surgical resection and radiation therapy, and even with recent use of immunotherapies, the five-year overall survival rate for OSCC patients remains at ~50% [1], largely due to late-stage diagnosis and metastasis of the primary tumor to the cervical lymph nodes [1]. A major driver of poor prognosis is the highly immunosuppressive tumor microenvironment (TME), which promotes tumor progression, impairs anti-tumor immune responses, and contributes to treatment resistance [2].

The OSCC TME is a dynamic and heterogeneous ecosystem composed of immune and stromal components, including cancer-associated fibroblasts (CAFs), tumor-associated macrophages (TAMs), T cells, and endothelial cells that collectively orchestrate processes such as immune evasion, angiogenesis, extracellular matrix remodeling, and epithelial–mesenchymal transition (EMT) [2]. Understanding the molecular mechanisms regulating the communication among the various TME cell types is critical for identifying novel therapeutic targets and improving clinical outcomes in OSCC.

PIAS1 (Protein Inhibitor of Activated STAT1) is a member of the SUMO (small ubiquitin-related modifier) E3 ligase family. PIAS1 binds selectively to specific substrate proteins, on the one hand, and the SUMO molecule, on the other, leading SUMO molecules to be conjugated via its C-terminal carboxy group to the epsilon amino side chain on a lysine residue within the substrate. In cancer, PIAS1 is involved in critical processes such as proliferation, apoptosis, DNA repair, and immune regulation [3]. Depending on tumor context, PIAS1 can exhibit either oncogenic or tumor-suppressive functions. For example, it promotes androgen receptor (AR) stability and tumor growth in prostate cancer [4]. Conversely, PIAS1 suppresses TGF-β-driven EMT in breast cancer via SUMOylation of SnoN [5]. These observations highlight the complexity of PIAS1’s role in cancer progression.

A majority of studies to date have focused on the role of PIAS1 in tumor cells, and a key but largely unstudied aspect of PIAS1 function is its role within the non-malignant cellular components of the tumor microenvironment (TME). To address this gap, we investigated PIAS1 expression and function across stromal and immune compartments in OSCC using patient-derived tissue microarrays (TMAs) and single-cell RNA sequencing (scRNA-seq). We found that PIAS1 is broadly expressed in the TME, but its expression is significantly reduced in CAFs, TAMs, T cells, and endothelial cells within tumors compared to their normal tissue counterparts. Differential gene expression analyses between PIAS1-positive and PIAS1-negative cells revealed cell-type-specific transcriptional signatures that may drive tumor progression and contribute to immune evasion. CellChat-based ligand–receptor modeling showed that PIAS1 reprograms intercellular communications toward anti-tumor signaling. These findings position PIAS1 as a critical modulator of the OSCC microenvironment, with potential utility as a prognostic biomarker and therapeutic target for reprogramming immune and stromal responses in cancer.

## 2. Material and Methods

### 2.1. Patient Cohort for TMA Analysis

TMAs were constructed from a cohort of 175 patients with histologically confirmed primary, surgically resected, treatment-naïve OSCC [6]. Clinical follow-up data, including treatment and outcomes, were updated as of 31 July 2024. The median patient age was 62.5 years, with a median follow-up duration of 5.8 years. Detailed clinical characteristics are summarized in Table 1. All research involving human tissue was performed following the guidelines outlined in the Tri-council Policy Statement for Research with Human Subjects (Canada). The study protocol was reviewed and approved by the Health Research Ethics Board of Alberta (HREBA) and followed the REMARK (Reporting Recommendations for Tumor Marker Prognostic Studies) guidelines for biomarker studies [7].

### 2.2. Fluorescence Immunohistochemistry for PIAS1

The protein abundance of PIAS1 was evaluated via fluorescence immunohistochemistry (IHC) using TMAs generated from the OSCC cohort. To account for inter-slide variability in staining intensity, each TMA slide included an on-slide control composed of cell lines with known differential PIAS1 expression. These PIAS1-positive controls enabled signal normalization of PIAS1 across the entire dataset using a reference TMA to generate a standard intensity curve, as described previously [8]. Antigen retrieval was performed in citrate-based buffer (Dako, S1699; Santa Clara, CA, USA) at 121 °C for three minutes using a decloaking chamber (Biocare Medical; Pacheco, CA, USA). Primary antibodies included a rabbit monoclonal anti-PIAS1 antibody (clone EPR2580(2), 1:2000, Abcam; Toronto, ON, Canada) and a mouse monoclonal anti-pan-cytokeratin antibody (clone AE1/AE3, 1:100, Dako). Isotype-matched controls were used to verify signal specificity. Secondary detection was achieved using anti-rabbit EnVision+ HRP-conjugated polymer (Dako, K4011; Santa Clara, CA, USA), and signal amplification was performed using TSA-Plus Cy5 reagents (PerkinElmer; Shelton, CT, USA). Nuclear staining was completed using DAPI (Thermo Fisher, D1306; Waltham, MT, USA), and slides were mounted with ProLong Gold anti-fade reagent (Thermo Fisher, P36934; Waltham, MT, USA), then stored at 4 °C until imaging. Antibody specificity was validated in-house through Western blotting and control tissue staining to confirm target specificity [8].

### 2.3. Quantitative Image Analysis

Digital images were analyzed as previously described, with all TMA slides digitized using the Aperio ScanScope FL (version 12.3.3, Aperio Inc., Vista, CA, USA) system under consistent image acquisition settings [8]. Quantification of the protein abundance of PIAS1 was performed using the HALO image analysis platform (version 2.0.1145.14, Indica Labs; Albuquerque, NM, USA). Tumor and stromal compartments were distinguished using masks derived from pan-cytokeratin staining. Specifically, a tumor-specific mask was created by thresholding the cytokeratin signal to distinguish epithelial tumor regions from the surrounding stroma. A stromal-specific mask was then defined as the inverse of the tumor mask, thereby capturing the non-epithelial compartment. Thresholding levels were optimized by spot-checking a representative subset of images to identify the most accurate cutoff values, which were subsequently applied uniformly across all samples. To ensure data quality, TMA cores were included in downstream analysis only if (i) more than 50% of the tissue was interpretable (i.e., free from artifacts such as folds, necrosis, or out of focus tissue) and (ii) more than 200 cells were detected per core. Following image review and validation of algorithm performance, only samples that had usable results were retained for subsequent statistical analysis [8].

### 2.4. ScRNAseq Data Acquisition and Sample Selection

The GSE181919 [9,10] dataset (Table 2 and Table 3) was used to investigate the mRNA abundance of PIAS1 in the TME. This dataset contains scRNA-seq data derived from HNSCC, including OSCC samples; both cancerous (CA) and normal (NL) tissues samples were profiled, allowing the comparative analysis of gene expression profiles across various cell types between tumor and normal samples.

Raw count matrices and corresponding cell-level metadata from GSE181919 [10] were downloaded from Gene Expression Omnibus (GEO) and loaded onto R. The gene-by-cell UMI count matrix (GSE181919_UMI_counts.txt) and barcode-level annotations (GSE181919_Barcode_metadata.txt) were read using the read.delim2() function. To ensure metadata alignment, column names in the count matrix were matched to the corresponding barcodes in the metadata. A Seurat object was created using the CreateSeuratObject() function, incorporating the barcode metadata and raw counts (set as the RNA assay) for downstream analysis [11]. To focus the analysis on HPV-negative cases, samples labeled as HPV-positive were excluded. Within the HPV-negative subset, primary OSCC (CA) samples (P4, P6, P7, P15, P21, P26, P30, P31, P51, P60) were selected. Additionally normal tissue from other HPV-negative head and neck sub-sites were also included. These two groups were merged using the merge() function to generate a dataset containing both normal and tumor samples. Seurat’s JoinLayers() function was then applied to combine the separate data layers (CA and NL) into a single layer within the default “RNA” assay [11,12]. The resulting object was saved for downstream processing.

To expand our analysis of the OSCC TME, we further incorporated cancer-only scRNA-seq data from two additional studies: GSE164690 [13] and GSE188737 [14] (Table 2, Table 4 and Table 5). These datasets were downloaded and processed using the same workflow as GSE181919. For consistency, only HPV-negative OSCC samples were retained.

### 2.5. Quality Control, Data Normalization, Integration, and Clustering

To characterize transcriptionally distinct cell populations across datasets, we applied a workflow including quality control, normalization, batch effect correction, unsupervised clustering, and cell type annotation using Seurat v5.2.1 [15].

To ensure high-quality cell representation, a quality control step was performed to remove damaged cells. Mitochondrial genes were identified by the prefix “MT-”, which indicates mitochondrial-encoded transcripts [16]. Using Seurat’s PercentageFeatureSet() function [16], the proportion of total transcripts derived from mitochondrial genes was calculated for each cell. Elevated levels of mitochondrial transcripts often indicate cellular damage, such as mitochondrial membrane permeabilization and are commonly associated with the initiation of apoptotic processes. To ensure data quality, cells were excluded from downstream analysis if they exhibited >15% mitochondrial transcript content [17].

To characterize transcriptionally distinct cell types independent of predefined annotations, we performed unsupervised clustering on both individual and integrated scRNA-seq data using Seurat. Each Seurat object was first independently normalized using the SCTransform() function, a scRNA-seq normalization method that applies a variance-stabilizing transformation while accounting for technical noise such as differences in sequencing depth and gene-specific expression variability across cells [16]. For example, expression of the housekeeping gene *GAPDH* may vary across epithelial cells due to differences in mRNA capture efficiency or sequencing depth.

Following normalization, datasets were integrated using Seurat’s shared nearest neighbor (SNN) PCA-based integration workflow, which identifies mutual anchors across datasets to align shared cell states. This approach corrects for batch effects introduced by sample origin, patient heterogeneity, and technical platforms, while preserving genuine biological differences [18,19].

Principal component analysis (PCA) was applied to the integrated object, and the top 30 principal components (PCs) were selected based on elbow plot inspection, a visual method used to determine the optimal number of PCs to retain. The “elbow” represents the point where the gain in explained variance begins to plateau, indicating that additional PCs capture minimal additional information [20]. A shared nearest neighbor (SNN) graph was then generated using FindNeighbors(), followed by visualization with RunUMAP() [18].

To determine the optimal clustering resolution, we applied Seurat’s FindClusters() function across a range of resolutions (0.01 to 1.00, in 0.01 increments). Clustering stability was assessed using the clustree package, which visualizes cluster transitions across resolutions. Stability scores from SC3 clustering were computed to quantitatively identify the resolution with the highest average cluster stability. The resolution yielding the highest SC3 score was selected for downstream analysis. Final clusters were assigned and visualized in Uniform Manifold Approximation and Projection (UMAP) space using a curated color palette [21]. In summary, the elbow plot helps determine how many principal components to use, ensuring key biological variation is captured while minimizing noise. Clustree and SC3 stability scores then use this variation to identify the clustering resolution that yields the most stable and meaningful cell clusters.

### 2.6. Differential Expression and Cell Type Annotation

To define transcriptional signatures for each cluster, we performed differential gene expression analysis using Seurat’s FindAllMarkers() function, with the RNA assay set as default [16]. Genes were retained if they were expressed in ≥ 25% of cells [22] and exhibited a log2 fold change ≥ 0.58 or ≤ −0.58, with an adjusted *p*-value ≤ 0.05. Cell-type-specific DEG lists were generated and used for manual annotation and downstream analyses (Data available). A curated marker gene list from CellTypist was used as a reference for manual annotation [23]. Annotation for major cell types, including cancer cells, CAFs, TAMs, and T cells, was confirmed using literature-validated canonical markers.

### 2.7. PIAS1 Expression Analysis Across Cell Types

Raw UMI count matrices were first normalized using Seurat’s NormalizeData() function, which applies log-normalization to standardize gene expression levels across cells [16]. This method adjusts for sequencing depth by dividing the UMI count for each gene by the total UMI count per cell, multiplying by a scale factor (default: 10,000), and applying a natural log transformation using the formula log(1 + x), where log is base e (~2.718) [24]. To assess PIAS1 expression across different cell types, log-normalized expression values for PIAS1 were extracted from the data matrix (slot = “data”). Corresponding cell-level metadata, including cell type annotations and tissue type (cancerous or normal), were also extracted.

A combined metadata table was generated, filtering out cell types with fewer than 200 cells and excluding those without comparable representation in both cancer and normal tissues. Twelve cell types remained after filtering, representing cancerous and normal counterparts (“Epithelial cell_NL”, “Cancer_CA”, “Fibroblast_NL”, “CAFs_CA”, “Endothelial cell_NL”, “Endothelial cell_CA”, “T cell_NL”, “T cell_CA”, “Macrophages_NL”, “TAM_CA”, “B cell_NL”, and “B cell_CA”). Custom cell type labels indicating the number of cells (n) per group were generated for plotting purposes.

To visualize PIAS1 expression across the selected cell types, a stacked bar plot was generated using the geom_bar (position = “stack”) function. Cells were classified as “Expressed” if they exhibited non-zero PIAS1 transcript counts or “Not Expressed” otherwise. The proportion of each expression status was calculated for each annotated cell type and visualized using the ggplot2 package (v3.5.2) [25].

To assess differences in PIAS1 expression within the PIAS1-expressing groups between matched normal (NL) and cancer (CA) populations, PIAS1-expressing cells were first extracted. Box plots were then generated using the geom_boxplot function from the ggplot2 package [26] to compare log-normalized PIAS1 expression across paired cell types. Statistical significance between normal and cancer counterparts was evaluated using the Wilcoxon rank-sum test, a non-parametric method that compares distributions between two independent groups without assuming that the data follow a normal distribution. *p*-values were calculated and visualized using the stat_compare_means() function from the ggpubr package (v 0.6.0), which performs the statistical test and annotates *p*-values directly onto the plots [27]. Summary statistics, including the median, mean, and interquartile range (25th and 75th percentiles), were calculated for PIAS1-expressing cells within each cell type and are displayed in Table 2.

### 2.8. Differential Expression Analysis Between PIAS1^+^ and PIAS1-Cells Across Cell Types

To evaluate transcriptional differences associated with PIAS1 expression within specific cell types, we performed differential gene expression analysis comparing PIAS1^+^ and PIAS1-single cells. The integrated Seurat object, filtered for cell types of interest, including T cells, macrophages, and fibroblasts, was used. The RNA assay was set as the default, and expression values from the “data” slot (log-normalized counts) were utilized. Within each cell type, cells with undetectable PIAS1 expression (expression value = 0) were assigned to the PIAS1-group. Among PIAS1-expressing cells (non-zero expression), only the top 25% of expressers, defined as those above the 75th percentile, were classified as PIAS1^+^. Cells with low but non-zero PIAS1 expression were excluded from the analysis to enhance the biological contrast between the PIAS1^+^ and PIAS1-groups, ensuring comparison between cells with clearly high expression and those with a complete absence of expression. This classification based on PIAS1 expression was stored under the PIAS1_subgroup metadata column and used for downstream comparisons.

Following group assignment, differential expression analysis was performed using the FindMarkers() function in Seurat, using the Wilcoxon rank-sum test [28]. DEGs were filtered based on a log2 fold change ≥ 0.58 or ≤ −0.58, with an adjusted *p*-value ≤ 0.05. Genes meeting these criteria were considered significantly differentially expressed. Gene symbol, average log2 fold changes, and adjusted *p*-values for significant genes were saved for downstream analyses (Tables S1–S3 Supplementary Materials).

### 2.9. Ingenuity Pathway Analysis (IPA)

To evaluate functional changes, significant DEGs for each cell type (CAFs, TAMs, T cells) were analyzed using Qiagen’s IPA platform to identify enriched “Diseases and Bio Functions”. Due to a limited number of DEGs in some comparisons, Canonical Pathways were not consistently significant and were excluded from further analysis. IPA results were imported using the importIPAenrichment() function into the multienrichjam R package (Version 0.0.57.9) [29]. Rather than a blind top-ranked selection, key pathways relevant to tumor biology and immune responses were manually selected for each cell type, and only these selected pathways were retained for visualization. Bar plots of activation z-scores were generated using ggplot2, with pathways separated by activation status (z-score > 0, “Activated”; z-score < 0, “Inhibited”) [30].

### 2.10. Cytokine Expression and Immune Checkpoint Analyses Between PIAS1^+^ and PIAS1-Cells

Gene set enrichment analyses (GSEA) were performed to evaluate the role of PIAS1 in cytokine regulation using anti-inflammatory cytokine panels. The anti-inflammatory cytokine panel included *CCL18*, *CCL19*, *CCL21*, *IL10*, *IL11*, *IL12A*, *IL12B*, *IL13*, *IL18*, *IL2*, *IL22*, *IL23A*, *IL24*, *IL4*, *IL6*, *TGFB2*, *TGFB1*, *IL1RN*, *IL37*, *IL27*, *IL35*, *IL20*, *IL33* [31]. To compare anti-inflammatory cytokine expression between PIAS1^+^ and PIAS1-cells GSEA was performed using the GSEA() function from the clusterProfiler package (v4.12.6). Enrichment plots were generated using the gseaplot2() function from the enrichplot package (v1.24.4) [32]

To assess the impact of PIAS1 expression on immune checkpoint regulation across tumor and major tumor microenvironmental compartments, a set of immune checkpoint genes was analyzed across PIAS1^+^ and PIAS1-cells. The checkpoint gene panels were defined specifically for each cell type based on the published literature. For T cells, the panel included PDCD1 (PD-1), CTLA4, LAG3, TIGIT, HAVCR2 (TIM-3), and BTLA (CD272) [33]. Cancer cells were evaluated for CD274 (PD-L1) [34] and CD276 (B7-H3) [35], while CAFs and TAMs were evaluated for CD276 [36,37]. Log-normalized expression values were extracted from the integrated Seurat object, and comparisons between PIAS1^+^ and PIAS1-cells were performed independently for each gene using Wilcoxon rank-sum tests. Results were visualized using bar plots (from the ggplot2 package) displaying log-normalized expression, and statistical significance was indicated using standard annotation (**** *p* < 0.0001, *** *p* < 0.001, ** *p* < 0.01, * *p* < 0.05, ns: not significant).

### 2.11. Cell–Cell Communication Analyses Between PIAS1^+^ and PIAS1^−^ Cells

To investigate how PIAS1 expression alters cell–cell communication within the OSCC microenvironment, we performed ligand–receptor interaction analysis between PIAS1^+^ and PIAS1-subgroups across CAFs, TAMs, and T cells using CellChat v1.6.1 [38]. Interactions probabilities were calculated using the computeCommunProb() function, and ranked using rankNet, focusing specifically on signaling between each PIAS1-defined subgroup and cancer cells in the integrated dataset. Differential interaction strength between PIAS1^+^ and PIAS1-cells was assessed using the compareInteractions() function, with statistical significance determined by Wilcoxon testing and further validated against a null distribution generated through permutation resampling (n = 1000) [39]. Permutation resampling involved randomly shuffling the PIAS1^+^ and PIAS1-labels 1000 times to simulate a scenario in which no real difference exists between the groups, thus generating a background (null) distribution. This distribution was used to compare the observed differences and evaluate whether they were greater than expected by chance. The top 10 most differentially regulated ligand–receptor pairs, ranked by the change in interaction probability (Δ), were visualized in both outgoing and incoming directions, capturing PIAS1-dependent shifts in intercellular signaling dynamics. The directionality of differential signaling is represented by color, red for interactions enriched in PIAS1^+^ cells and blue for those enriched in PIAS1-cells. Ligand–receptor functions were annotated using CellChat’s curated signaling database, which uses expert-reviewed literature and functional pathway data, and were further validated using the recent literature where available.

## 3. Results

### 3.1. Stromal PIAS1 Expression Correlates with Improved Survival in OSCC

To investigate the prognostic relevance of PIAS1 in OSCC, we evaluated its protein abundance using fluorescence IHC of patient-derived TMAs. The sensitivity and specificity of the PIAS1 antibody were previously validated [8]. Slide-to-slide staining variability was normalized using on-slide control cell lines with defined PIAS1 expression levels. A single reference TMA was used to align all standard curves, allowing normalization across TMAs.

**Figure 1:**
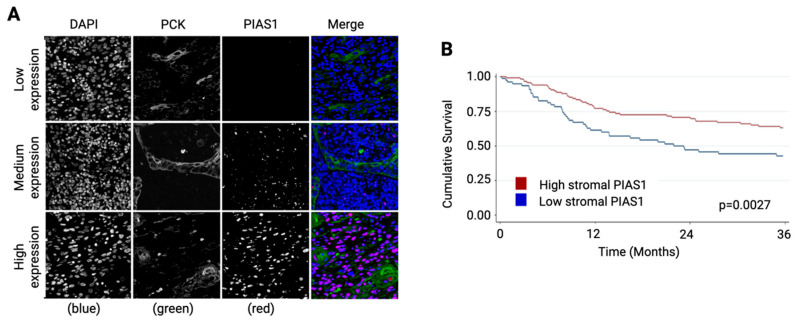
Fluorescence immunohistochemistry analysis of OSCC TMAs. (**A**) Representative immunofluorescence images of stromal PIAS1 staining in OSCC patient samples from the Ohlson cohort, demonstrating DAPI-stained nuclei (blue), anti-pan-cytokeratin-stained tumor epithelial cells (green), and anti-PIAS1 staining (red). (**B**) Kaplan–Meier curve comparing overall survival (OS) between patients with high versus low stromal PIAS1 expression, dichotomized at the median.

Pan-cytokeratin staining was used to distinguish epithelial and stromal regions, and patients were stratified into high and low stromal PIAS1 groups based on staining intensity (Figure 1A). Kaplan–Meier analysis revealed that patients with high stromal PIAS1 expression were associated with significantly improved overall survival (*p* = 0.0027; Figure 1B), suggesting a tumor-suppressive role of PIAS1 in the OSCC TME.

### 3.2. Single-Cell Profiling Reveals Heterogeneity in Immune and Stromal Cells in the OSCC TME

**Figure 2:**
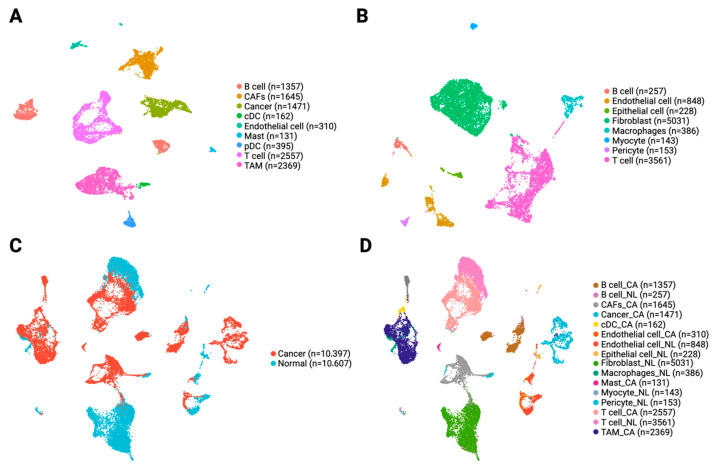
Single-cell RNAseq analysis of OSCC samples. UMAP visualization of cells from (**A**) OSCC tissues, indicating assigned cell types; (**B**) normal tissues, indicating assigned cell types; (**C**) integrated normal (in blue) and OSCC tissue (in red); (**D**) integrated normal and OSCC data, indicating assigned cell type.

To explore the cellular landscape of PIAS1 expression, we analyzed scRNA-seq data from primary OSCC and normal tissues. We identified major cell populations within OSCC samples including cancer epithelial cells, CAFs, TAMs, T cells, B cells, endothelial cells, conventional dendritic cells (cDCs), plasmacytoid dendritic cells (pDCs), and mast cells (Figure 2A). Normal tissues, on the other hand, contained fibroblasts, endothelial cells, T cells, B cells, pericytes, macrophages, myocytes, and epithelial cells as the major cell types (Figure 2B).

As expected, several stromal and immune populations, such as endothelial cells, T cells, and B cells, were shared between normal and cancer tissues, while CAFs and TAMs were exclusively detected in OSCC samples, consistent with their established roles in tumor progression and immune modulation. We note that some stromal and immune cell populations in our dataset are challenging to distinguish with absolute certainty based on single-cell transcriptomes alone. Specifically, CAFs and fibroblasts share many canonical markers, while TAMs and tissue-resident macrophages also display substantial overlap in commonly used markers. In this study, CAFs and TAMs were annotated based on their enrichment in tumor samples, co-expression of genes frequently associated with tumor-promoting phenotypes (e.g., ACTA2, FAP, and COL11A1 for CAFs [40]; CD163, MSR1, and CD206 as typical M2 macrophage markers [41]), along with their absence in normal tissues. However, because these gene expression patterns are not entirely specific, some cells classified as CAFs or TAMs may represent activated counterparts from adjacent normal tissue.

Notably, normal epithelial cells formed small, cohesive clusters, while cancer epithelial cells displayed broader transcriptional heterogeneity in UMAP visualization (Figure 2C,D and Supplementary Figure S1A–C). This integrated dataset, containing both cancer-derived and normal TME cells, serves as a foundation for further investigating PIAS1-associated transcriptional changes within specific stromal populations. It also enables direct comparisons between tumor-associated and normal stromal counterparts in OSCC.

### 3.3. PIAS1 Expression Is Broadly Reduced in the OSCC TME and Associated with Tumor-Suppressive Programs

**Figure 3:**
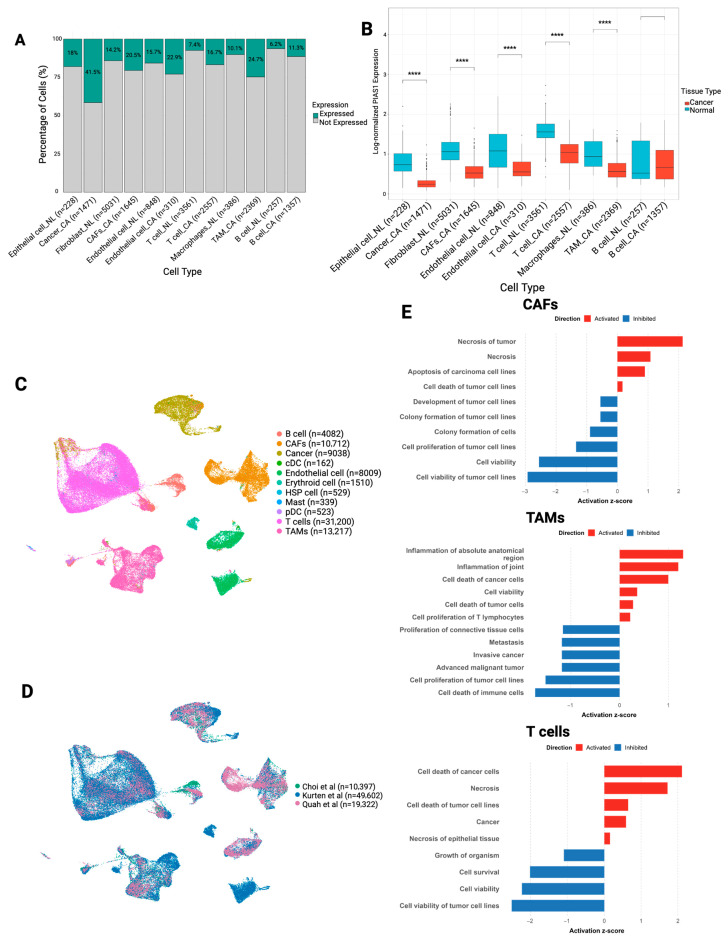
Transcriptomic differences between PIAS1^+^ versus PIAS1-cells in the TME. (**A**) Bar plot showing the percentage of cells expressing PIAS1 across major cell types in normal (NL) and OSCC (CA) tissues. (**B**) Boxplots comparing normalized PIAS1 expression levels between normal and cancer-associated cells across major cell types. Statistical significance was assessed using the Wilcoxon rank-sum test (****, adjusted *p* < 0.0001). (**C**) UMAP visualization of integrated single-cell RNA-seq data from HPV-negative OSCC samples across three cohorts (Choi [10], Kürten [13], Quah [14]), colored by cell type. (**D**) UMAP visualization of integrated single-cell RNA-seq data from HPV-negative OSCC samples across three cohorts (Choi [10], Kürten [13], Quah [14]), colored by study cohort. (**E**) Ingenuity Pathway Analysis (IPA) “Diseases and Bio Functions” enriched in differentially expressed genes between PIAS1^+^ and PIAS1-cells within CAFs, TAMs, and T cells from the integrated data. Displaying top activated (positive z-scores, red) and top inhibited (negative z-scores, blue) biological functions per cell type.

We next quantified PIAS1 expression across matched stromal and immune cell types from normal and OSCC tissue. Cells were classified as PIAS1-expressing if they exhibited a detectable nonzero transcript count. Across all analyzed cell type pairs, including malignant epithelial cells, TAMs, and CAFs, a higher proportion of cells expressed PIAS1 in tumors compared to normal tissue (Figure 3A). However, within PIAS1-expressing cells, we observed significant reduction in PIAS1 expression across almost all cancer-associated cell types compared to their normal counterparts (Figure 3B; Wilcoxon rank-sum test, adjusted *p* < 0.0001). Notably, B cells were the only population in which PIAS1 expression remained relatively unchanged. PIAS1 expression levels in TME cell types across cancer and normal tissue are summarized in Table 6.

To investigate the functional role of PIAS1 within the TME, we focused on scRNA-seq data from three independent cohorts of HPV-negative OSCC [10,11,12]. Canonical marker heatmaps confirmed accurate cell-type annotation (Supplementary Figure S2A). UMAP visualization of the integrated dataset revealed clear clustering by cell type (Figure 3C) and demonstrated successful integration across studies (Figure 3D), supporting the robustness of downstream analyses (Supplementary Figure S2B,C).

Differential gene expression analysis (DEA) between PIAS1^+^ and PIAS1-cells within each population revealed distinct, cell-type-specific transcriptional signatures (Figure 3E). IPA indicated that PIAS1^+^ CAFs were enriched in pathways involved in tumor necrosis and apoptosis, along with suppression of functions related to tumor growth. In TAMs, PIAS1 expression correlated with tumor cell death and inflammatory functions, while functions associated with immune cell death, tumor proliferation, invasion, and metastasis were among the most strongly inhibited. PIAS1^+^ T cells exhibited significant reduction in tumor cell viability and activation of cell death pathways.

Collectively, these data suggest that although more cells in OSCC express PIAS1, expression levels per positive are attenuated in key cell types. High PIAS1 expression within individual cells is associated with tumor-suppressive transcriptional programs across the OSCC TME.

### 3.4. PIAS1 Limits Immune Escape and Immune Checkpoint Expression

**Figure 4:**
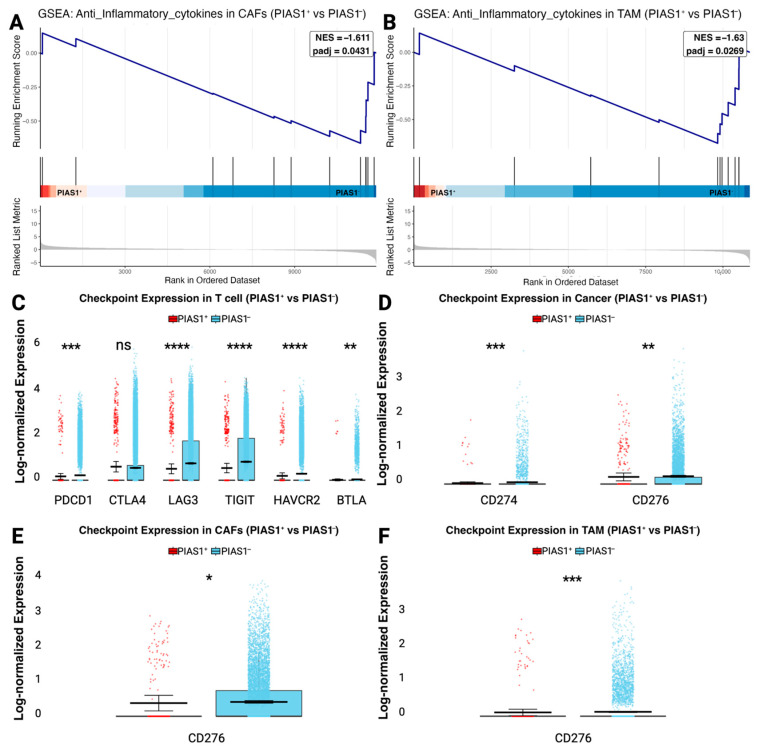
Gene set enrichment analysis of cytokine and checkpoint protein encoding genes in PIAS1^+^ versus PIAS1-cells in the TME. Gene set enrichment analysis (GSEA) demonstrating enrichment of anti-inflammatory cytokines in (**A**) CAFs and (**B**) TAMs comparing PIAS1^+^ and PIAS1-cells from tumor samples. Immune checkpoint gene expression comparisons between PIAS1^+^ and PIAS1–cells across T cells (**C**), cancer cells (**D**), CAFs (**E**), and TAMs (**F**). Statistical significance was assessed using Wilcoxon rank-sum test: **** *p* < 0.0001, *** *p* < 0.001, ** *p* < 0.01, * *p* < 0.05, ns = not significant.

To further characterize the immunoregulatory role of PIAS1 in the TME, we examined its association with cytokine signaling and immune checkpoint expression across major stromal and immune components. GSEA using curated gene sets demonstrated significant enrichment of anti-inflammatory cytokine pathways in PIAS1-CAFs and TAMs (Figure 4A,B), indicating that loss of PIAS1 is associated with a more immunosuppressive, anti-inflammatory transcriptional profile. No significant enrichment was observed in T cells.

We next compared the expression of immune checkpoint molecules between PIAS1^+^ and PIAS1-cells across tumor-infiltrating T cells (Figure 4C), cancer cells (Figure 4D), CAFs (Figure 4E), and TAMs (Figure 4F). Across all cell types, immune checkpoint gene expression was significantly upregulated in PIAS1-cells, suggesting PIAS1 suppresses immune checkpoint activation. These position PIAS1-positive cells as a regulator of immune evasion by restricting anti-inflammatory signaling and immune checkpoint expression in the OSCC TME.

### 3.5. PIAS1 Modulates Cell–Cell Communication and Supports Anti-Tumor Immunity

To determine whether PIAS1 expression alters intercellular communication, we analyzed ligand–receptor interactions between PIAS1^+^ or PIAS1-CAFs, TAMs, and T cells and cancer cells using CellChat.

**Figure 5:**
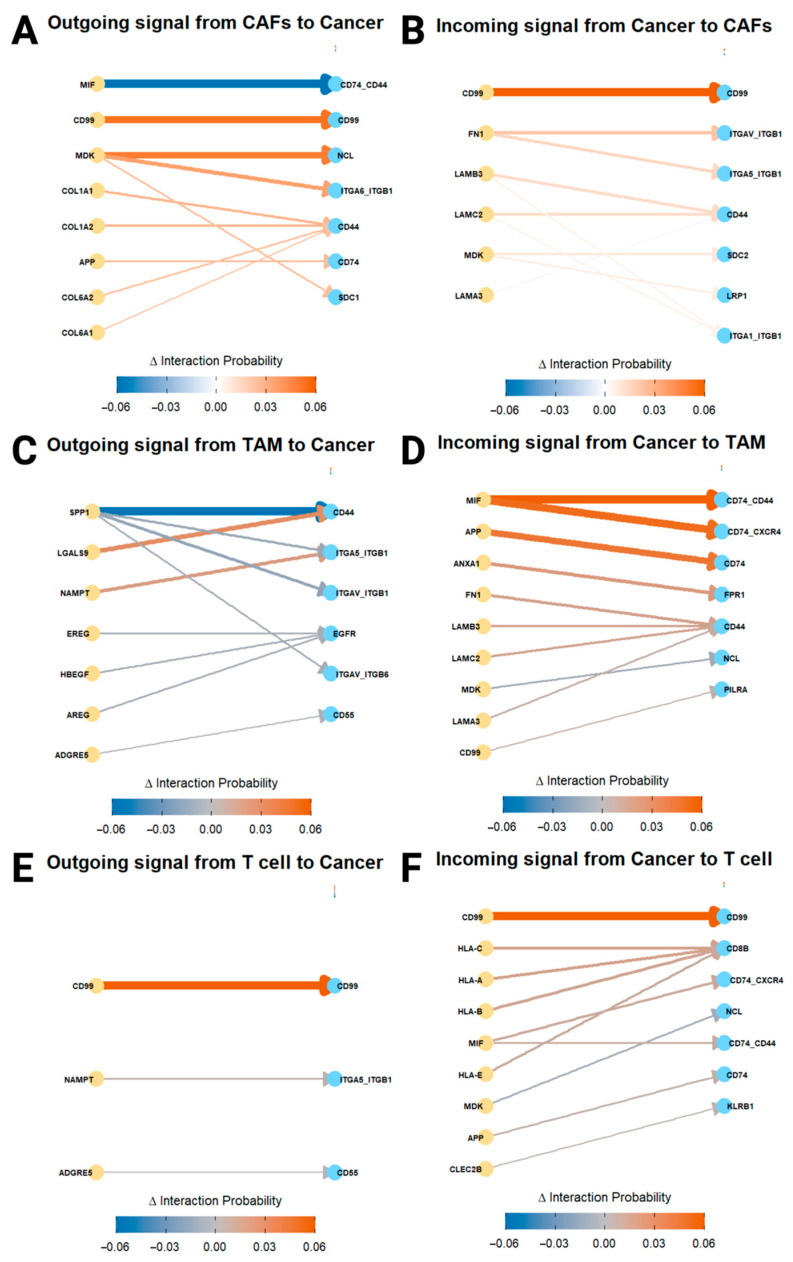
Cell–cell communication analysis between cancer cells and other cells in the TME stratified by PIAS1 expression. (**A**) Outgoing signals from CAFs to cancer cells. (**B**) Incoming signals to CAFs from cancer cells. (**C**) Outgoing signals from TAMs to cancer cells. (**D**) Incoming signals to TAMs from cancer cells. (**E**) Outgoing signals from T cells to cancer cells. (**F**) Incoming signals to T cells from cancer cells. Arrows represent ligand–receptor pairs, with directionality pointing toward the receiver cell type. Line thickness reflects the magnitude of the interaction change (Δ Interaction Probability), with red lines indicating increased interactions in PIAS1^+^ cells and blue lines indicating increased interactions in PIAS1-cells.

In PIAS1^+^ CAFs, ligand–receptor interactions revealed a shift toward a less tumor-promoting phenotype. Most notably, MIF–CD74_CD44 signaling, known to induce immune suppression and matrix metalloproteinase (MMP)-mediated tumor invasion, was markedly downregulated [42,43]. Conversely, CD99–CD99 interactions were upregulated in both incoming and outgoing directions, consistent with tumor-suppressive roles of CD99 [44], while MDK–NCL and MDK–ITGA6_ITGB1 interactions, typically linked to CAF activation and cancer cell adhesion [45,46], were also elevated in PIAS1^+^ CAFs; these may have context-dependent functions in OSCC. Modest increases in ECM-related interactions such as COL1A1/COL1A2–CD44 and COL6A1/COL6A2–CD44 likely reflect matrix maintenance rather than pro-invasive remodeling (Figure 5A).

Incoming signals from cancer cells to PIAS1^+^ CAFs were subtly altered, including integrin and laminin-mediated adhesion pathways (e.g., FN1–ITGAV_ITGB1, FN1–ITGA5_ITGB1, LAMB3–CD44 and LAMC2–CD44), which support basement membrane integrity rather than invasion [47] (Figure 5B). Collectively, these changes suggest that PIAS1 expression in CAFs reprograms the stromal compartment toward a less immunosuppressive, more quiescent phenotype.

PIAS1^+^ TAMs exhibited a reduction in pro-tumorigenic signaling, including strong downregulation of SPP1–CD44 interaction, a pathway that promotes M2 polarization [48]. Multiple SPP1–integrin interactions (e.g., SPP1–ITGA5_ITGB1, SPP1–ITGAV_ITGB1, and SPP1–ITGAV_ITGB6) were similarly suppressed [49] (Figure 5C). In contrast, LGALS9–CD44 and NAMPT–ITGA5_ITGB1, were moderately upregulated in PIAS1^+^ TAMs. Although LGALS9 (Galectin-9) has been classically linked to T cell exhaustion [50], it has also been shown to inhibit metastasis through endothelial adhesion blockade [51]. NAMPT, a metabolic regulator, showed only a moderate upregulation in PIAS1^+^ TAMs with unclear biological significance [52] (Figure 5C).

Incoming signals to PIAS1^+^ TAMs from tumor cells included elevated MIF–CD74_CD44 and MIF–CD74_CXCR4 interactions, which in this context may promote M1 polarization [43,53]. Other increased interactions included APP–CD74, linked to modulation of phagocytosis in macrophages [54], and ANXA1–FPR1, associated with macrophage recruitment [55]. ECM inputs including FN1–CD44, LAMB3–CD44 and LAMC2–CD44 were again modestly increased (Figure 5D). Together, PIAS1^+^ TAMs exhibited a signaling profile favoring reduced M2 polarization and immune activation.

In PIAS1^+^ T cells, CD99–CD99 emerged as the dominant upregulated outgoing signal to cancer cells (Figure 5E). CD99 is known to promote T cell infiltration into tumor and co-stimulatory activation, potentially contributing to an immune-hot environment [56] (Figure 5E). This signal was reciprocally enhanced in the incoming direction as well. Additionally, moderate increases in HLA class–CD8B interactions suggested enhanced tumor antigen presentation and activation of cytotoxic T cell responses [57] (Figure 5F).

## 4. Discussion

This study identifies PIAS1 as a cell-type-specific modulator of the tumor microenvironment (TME) in OSCC, with functional consequences for tumor progression, immune regulation, and clinical outcomes. By combining immunohistochemical analysis of patient-derived tissue microarrays (TMAs) with publicly available single-cell transcriptomic data, we demonstrate that high stromal PIAS1 expression is associated with improved patient survival and that PIAS1 governs distinct, tumor-suppressive transcriptional programs across key stromal and immune cell types within the OSCC TME.

High stromal PIAS1 protein levels were significantly associated with better overall survival, suggesting that PIAS1 may influence patient outcome through modulation of the TME rather than acting solely within malignant cells. Although previous studies have implicated PIAS1 as a suppressor of TGF-β signaling and EMT in breast cancer [5,8,58,59], its prognostic relevance in OSCC has not been clearly established. A recent pan-cancer analysis across 33 cancer types noted tissue-specific variability in PIAS1 expression [60], but did not specifically examine OSCC.

Analysis of publicly available scRNA-seq datasets revealed that while a greater proportion of stromal and immune cells express PIAS1 in tissue tumors relative to normal tissue, per-cell expression levels were significantly reduced in CAFs, TAMs, T cells, and endothelial cells. This reduction in expression within PIAS1-positive cells suggests a potential functional silencing or repression of PIAS1 activity within the tumor milieu. This attenuated expression may contribute to immune evasion and tumor progression.

Single-cell analysis across three independent OSCC cohorts revealed that PIAS1^+^ stromal and immune cells exhibit cell-type-specific transcriptional programs associated with tumor suppression. In CAFs, TAMs and T cells, IPA identified activation of pathways promoting tumor cell death, inhibition of tumor proliferation, and suppression of metastatic behavior. Notably, the reduced PIAS1 expression in OSCC may impair these tumor-suppressive pathways. By contrast, PIAS1-cells across these lineages showed enrichment for anti-inflammatory signaling and immune checkpoint expression, suggesting a shift toward a more immunosuppressive state. Consistent with reports that PIAS1 can inhibit Treg differentiation by repressing *Foxp3* promoter accessibility [61], we observed that PIAS1-CAFs and TAMs were enriched for anti-inflammatory cytokine pathways, which is indicative of a more immunosuppressive transcriptional landscape.

While direct studies on PIAS1 in the TME are limited, our findings align with its known role in regulating TGF-β signaling, a central pathway in establishing immunosuppressive microenvironments. In OSCC, TGF-β promotes fibroblast activation, ECM remodeling, M2 polarization of macrophages, T cell exhaustion, and immune checkpoint induction [2,61,62,63,64]. PIAS1 may therefore exert anti-tumor effects, in part, by restraining TGF-β-driven immunosuppression [5,8,58,59,61].

Moreover, our ligand–receptor interaction analyses revealed that PIAS1-positive stromal and immune cells engage in signaling networks consistent with immune activation. In CAFs, PIAS1^+^ cells showed downregulation of immunosuppressives MIF–CD74_CD44 signaling and upregulation of tumor-suppressive CD99–CD99 interactions associated with immune activation. In TAMs, SPP1–CD44 and related integrin signals, key mediators of M2 polarization, were suppressed in PIAS1^+^ cells, while tumor-derived MIF signals promoting M1-polarizing were enhanced. In T cells, PIAS1^+^ subsets uniquely upregulated CD99–CD99 and MHC-I–CD8 interactions, thus promoting T cell infiltration, antigen presentation, and effector activation.

Together, our findings position PIAS1 as a multifunctional regulator of the OSCC TME, restraining both stromal support for tumor progression and immune escape. The consistent association between stromal PIAS1 expression and favorable biological features, including tumor cell death, pro-inflammatory signaling, and reduced immune checkpoint expression, supports its role as a critical orchestrator of an immune-active microenvironment. These observations underscore PIAS1’s potential as both a prognostic biomarker and a target for stromal reprogramming strategies in OSCC. PIAS1 modulates cell–cell interactions and transcriptional states across CAFs, TAMs, and T cells, collectively promoting a tumor-suppressive TME. Future studies should aim to elucidate upstream regulators of PIAS1 and determine whether restoring or enhancing its activity can reinvigorate anti-tumor immunity and sensitize tumors to immunotherapy. Such approaches could extend beyond OSCC to other solid tumors characterized by immune exclusion and stromal-driven resistance.

Despite the integrative nature of our study, several limitations should be noted. Our findings are derived from retrospective tissue-based analyses and computational modeling of single-cell transcriptomic datasets. While these approaches provide valuable insights into the TME, they lack direct functional validation. We did not perform in vitro or in vivo experiments to confirm the causal role of PIAS1 in shaping stromal or immune cell behavior. Future studies employing genetic knockdown or knockout models of PIAS1 in CAFs, tumor-associated macrophages TAMs, or immune cell co-culture systems will be essential to substantiate the mechanistic hypotheses proposed here. Additionally, our reliance on publicly available datasets introduces potential variability, in spite of our best efforts in correcting batch effects, due to differences in sample preparation, sequencing depth, and patient cohorts. Addressing these gaps through experimental validation and larger, independent patient cohorts would strengthen the translational implications of our results.

We focused on HPV-negative OSCC to reduce biological heterogeneity, as HPV-positive head and neck cancers are recognized as a distinct clinical and molecular entity with improved prognosis, greater treatment sensitivity, and markedly different immune microenvironments. Given that HPV-driven tumors often exhibit a more immune “hot” TME, with abundant CD8^+^ T cell and reduced M2 macrophage infiltration and elevated PD-L1 expression [65,66], the role of PIAS1 might differ significantly in this context. Future studies directly comparing PIAS1 function across HPV-positive and HPV-negative OSCC are warranted to determine whether it operates as a universal regulator of anti-tumor immunity or displays subtype-specific effects.

## 5. Conclusions

Our study defines PIAS1 as a pivotal regulator of the OSCC tumor microenvironment, linking high stromal expression to improved patient outcomes and coordinated anti-tumor immune signaling. Through integrative analysis of tissue microarrays and single-cell RNA-seq datasets, we demonstrate that PIAS1 modulates stromal and immune cell phenotypes, suppresses immune checkpoint pathways, and enhances pro-inflammatory transcriptional programs. Biologically, PIAS1 emerges as a suppressor of stromal immunosuppression in CAFs and TAMs. Translationally, these insights reveal new therapeutic opportunities: targeting PIAS1-regulated pathways or augmenting PIAS1 activity could complement existing immunotherapies and guide the development of novel, precision-based strategies to reprogram the OSCC TME.

## Figures and Tables

**Table 1:** Clinicopathological features of the Ohlson OSCC tissue microarray cohort (n = 175), including patient demographics, tumor staging, and relevant clinical parameters.

Variable	n (%) Unless Otherwise Indicated
Number	175
Sex	
*Female*	65 (37.1)
*Male*	110 (62.9)
Median age at diagnosis, years (IQR)	62.5 (53.9–74.8)
Pathologic T stage	
*T1/T2*	105 (60.0)
*T3/T4*	62 (35.4)
*Missing*	8 (4.6)
Pathologic N stage	
*Node-negative*	63 (36.0)
*Node-positive*	79 (45.1)
*Missing*	33 (18.9)
Overall Stage	
*Stage I/II*	61 (34.9)
*Stage III/IV*	107 (61.1)
*Missing*	7 (4.0)

**Table 2:** Summary of publicly available single-cell RNA sequencing datasets used for analysis in this study. Each dataset includes the accession number, study title, sequencing platform, number of patient samples and total cells profiled, and cancer type.

Dataset Accession	Study Title	Platform	No. of Samples/Cells	Cancer Type
GSE181919	Single-cell transcriptome profiling of the stepwise progression of head and neck cancer	10X Genomics Chromium	23/54,239	HNSCC
GSE164690	Investigating immune and non-immune cell interactions in head and neck tumors by single-cell RNA sequencing	10X Genomics Chromium	18/134,606	HNSCC
GSE188737	Single cell analysis in head and neck cancer reveals potential immune evasion mechanisms during early metastasis	10X Genomics Chromium	7/53,459	HNSCC

**Table 3:** Clinical characteristics of OSCC patients included in the scRNAseq study (GSE181919). Information includes patient ID, HPV status, anatomical subsite of the tumor and TNM staging, along with corresponding sample IDs.

Patient ID	HPV Status	Subsite	TN Stage
P4	Negative	Oral cavity	T2N2a
P6	Negative	Oral cavity	T2N1
P7	Negative	Oral cavity	T2N0
P15	Negative	Oral cavity	T1N0
P21	Negative	Oral cavity	T4aN1
P26	Negative	Oral cavity	T2N0
P30	Negative	Oral cavity	T2N0
P31	Negative	Oral cavity	T2N2
P51	Negative	Oral cavity	T2N1
P60	Negative	Oral cavity	T4aN0

**Table 4:** Clinical characteristics of OCSCC patients from the GSE164690 single-cell RNA sequencing dataset. Information includes patient ID, HPV status, anatomical subsite of the tumor, TN staging.

Patient ID	HPV Status	Subsite	TN Stage
1	Negative	Oral cavity	T4aN2b
2	Negative	Oral cavity	T3N2a
3	Negative	Oral cavity	T4aN0
4	Negative	Oral cavity	T3N1
5	Negative	Oral cavity	T3N3b
6	Negative	Oral cavity	T3N0
8	Negative	Oral cavity	T1N0
9	Negative	Oral cavity	T3N2b
10	Negative	Oral cavity	T3N0
11	Negative	Oral cavity	T2N0
15	Negative	Oral cavity	T2N0
22	Negative	Oral cavity	T4aN2c

**Table 5:** Clinical characteristics of OCSCC patients from the GSE188737 single-cell RNA sequencing dataset. Information includes patient ID, HPV status, anatomical subsite of the tumor, TN staging.

Patient ID	HPV Status	Subsite	TN Stage
HN237	Negative	Oral cavity	T4aN1
HN242	Negative	Oral cavity	T3N3b
HN251	Negative	Oral cavity	T3N3b
HN257	Negative	Oral cavity	T4aN3b
HN263	Negative	Oral cavity	T3N2bMx
HN272	Negative	Oral cavity	T4aN2b

**Table 6:** Summary statistics of PIAS1 expression across PIAS1-positive cells in matched normal and cancer-associated cell type pairs. Values represent the median, mean, and interquartile range (25th–75th percentiles) of log-normalized PIAS1 expression for each cell population. n Cells indicates the number of cells exhibiting nonzero PIAS1 expression within each cell population.

Cell Type	n Cells	Median	Mean	IQR (25th–75th Percentile)
Epithelial cell (NL)	41	0.734	0.830	0.569–1.010
Cancer (CA)	610	0.244	0.279	0.171–0.335
Fibroblast (NL)	712	1.060	1.100	0.853–1.300
CAFs (CA)	337	0.525	0.563	0.390–0.689
Endothelial cell (NL)	133	1.080	1.110	0.669–1.500
Endothelial cell (CA)	71	0.552	0.630	0.451–0.804
T cell (NL)	263	1.560	1.570	1.410–1.760
T cell (CA)	428	1.040	0.993	0.774–1.240
Macrophage (NL)	39	0.937	1.010	0.689–1.320
TAM (CA)	586	0.560	0.616	0.420–0.770
B cell (NL)	16	0.521	0.807	0.381–1.330
B cell (CA)	154	0.664	0.736	0.372–1.100

## Data Availability

Links to all publicly available datasets are provided within the manuscript. The TMA analysis data will be made available upon reasonable request.

## Supplementary Materials

The following supporting information can be downloaded at https://www.mdpi.com/article/10.3390/cancers17172905/s1, Supplementary Figure S1: (A) UMAP visualization of cells from OSCC tissues, colored by Patient ID. (B) UMAP visualization of cells from normal tissues, colored by Patient ID. (C) UMAP visualization of the integrated normal and OSCC data, with cells colored by Patient ID. Supplementary Figure S2: (A) Heatmap of canonical marker genes confirming major cell type identities across cancer cells, CAFs, T cells, and TAMs. (B) UMAP visualization of the integrated dataset before batch effect correction, with cells colored by Patient ID. (C) UMAP visualization of the integrated dataset after batch effect correction, with cells colored by Patient ID. Supplementary Table S1: DEGs between PIAS1+ and PIAS1− cells in T cells from integrated dataset and their fold change (log2 scale) and adjusted *p*-value (padj). Supplementary Table S2: DEGs between PIAS1+ and PIAS1− cells in macrophages from integrated dataset and their fold change (log2 scale) and adjusted *p*-value (padj). Supplementary Table S3: DEGs between PIAS1+ and PIAS1− cells fibroblasts from integrated dataset and their fold change (log2 scale) and adjusted *p*-value (padj).

## Abbreviations

The following abbreviations are used in this manuscript:

CA	Cancer Tissue
CAFs	Cancer-Associated Fibroblasts
CD	Cluster of Differentiation (used in various CD markers)
DEGs	Differentially expressed gene(s)
ECM	Extracellular Matrix
EMT	Epithelial–Mesenchymal Transition
FN1	Fibronectin 1
GEO	Gene Expression Omnibus
GSEA	Gene set enrichment analysis
HNC	Head and Neck Cancer
HNSCC	Head and neck squamous cell carcinoma
HREBA	Health Research Ethics Board of Alberta
HRP	Horseradish Peroxidase
IHC	Immunohistochemistry
IPA	Ingenuity Pathway Analysis
ITGA5/ITGB1	Integrin alpha-5/Integrin beta-1
MIF	Macrophage Migration Inhibitory Factor
NL	Normal Tissue
OSCC	Oral Squamous Cell Carcinoma
PCA	Principal component analysis
PIAS1	Protein Inhibitor of Activated STAT1
REMARK	Reporting Recommendations for Tumor Marker Prognostic Studies
RNA-seq	RNA sequencing
SC3	Single-cell Consensus Clustering
SNN	Shared Nearest Neighbor
SUMO	Small Ubiquitin-Related Modifier
TAMs	Tumor-Associated Macrophages
TMA	Tissue Microarray
TME	Tumor Microenvironment
UMAP	Uniform manifold approximation and projection
UMI	Unique Molecular Identifier
cDCs	Conventional Dendritic Cells
pDCs	Plasmacytoid Dendritic Cells
scRNA-seq	Single-cell RNA sequencing
Δ (Delta)	Change in Interaction Probability
