# Supplementary material for: PIAS1 Shapes a Tumor-Suppressive Microenvironment by Suppressing Immune Evasion in Oral Squamous Cell Carcinoma
Source: Cancers (Basel). 2025 Sep 4;17(17):2905. doi: 10.3390/cancers17172905 (PMC12427710; doi:10.3390/cancers17172905)
Supplement: Supplementary file 1 [file cancers-17-02905-s001.zip › cancers-3790809-supplementary.pdf]

cancers

MDPI

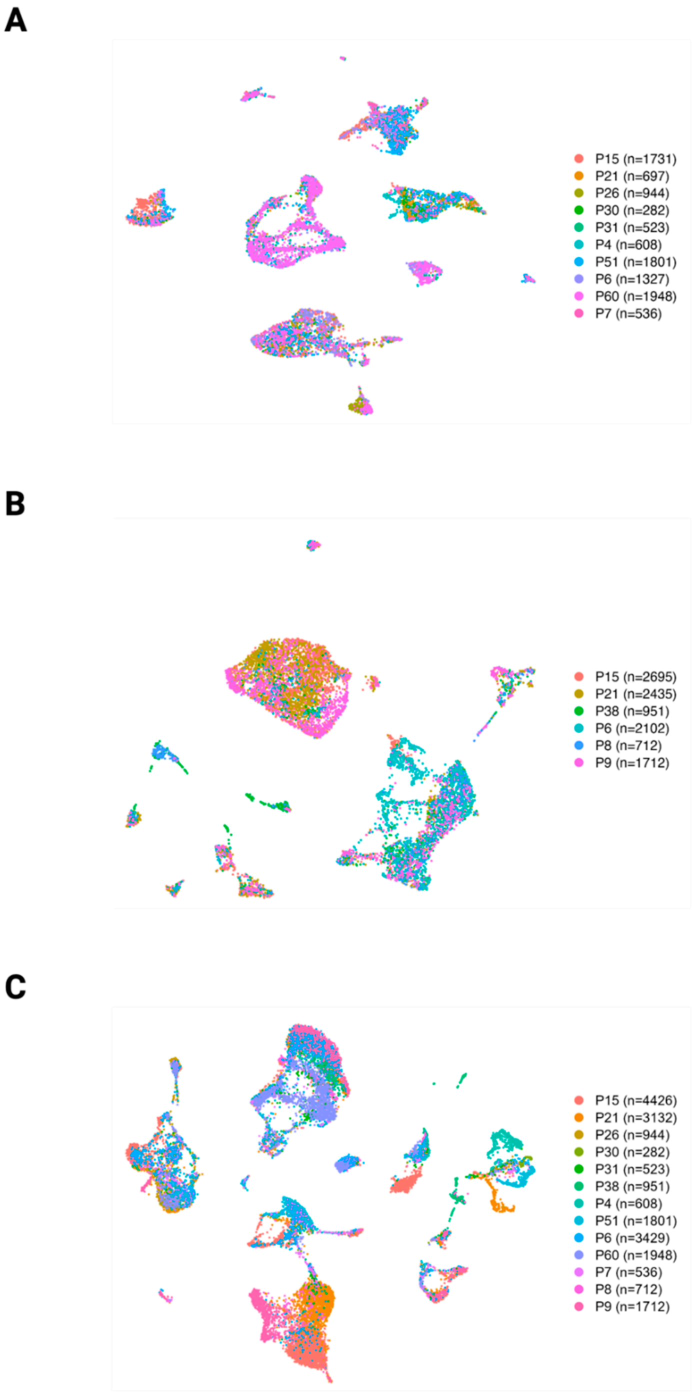

Supplementary Figure 1:
(A) UMAP visualization of cells from OSCC tissues, colored by Patient ID.
(B) UMAP visualization of cells from normal tissues, colored by Patient ID.
(C) UMAP visualization of the integrated normal and OSCC data, with cells colored by Patient ID.

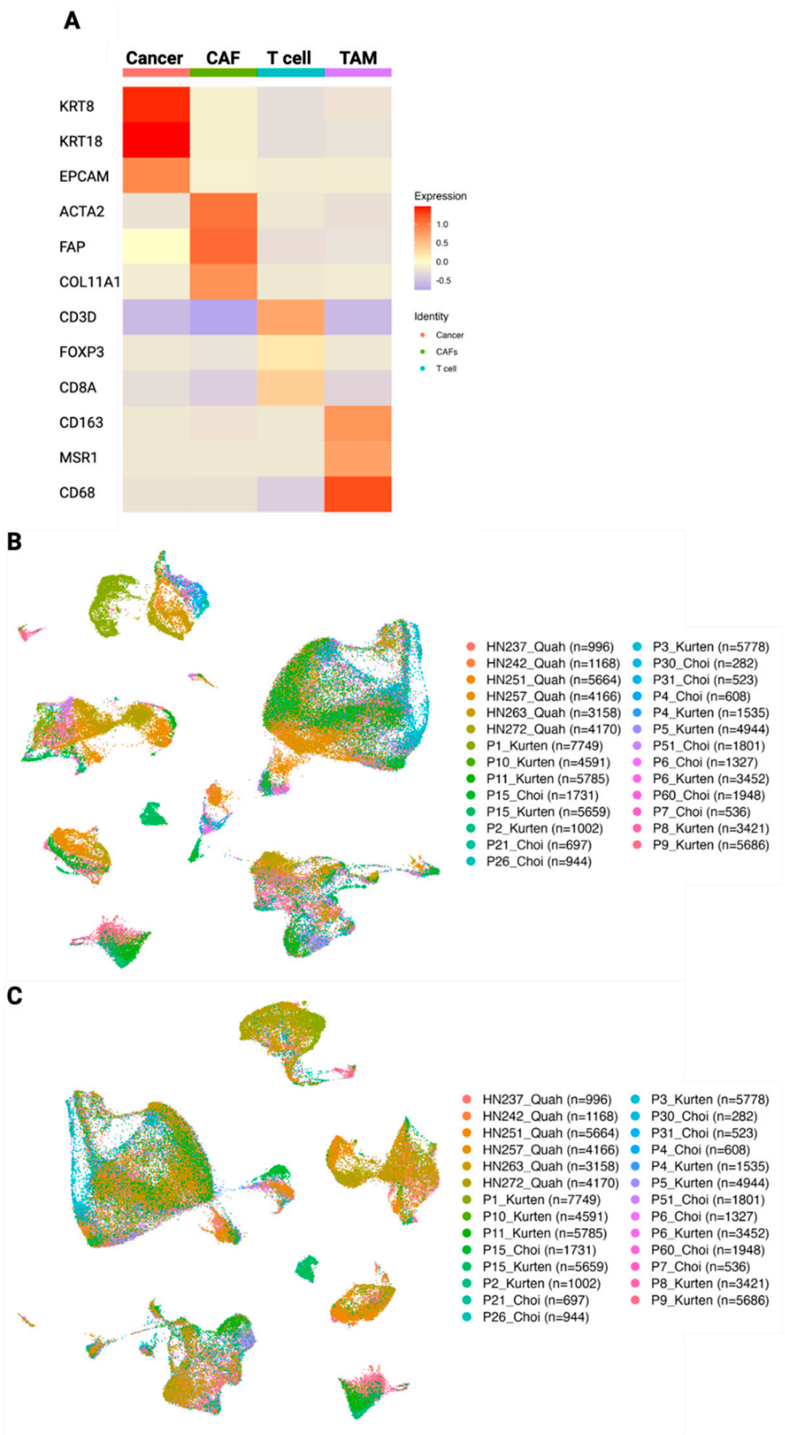

Supplementary Figure 2:
(A) Heatmap of canonical marker genes confirming major cell type identities across cancer cells, CAFs, T cells, and TAMs.
(B) UMAP visualization of the integrated dataset before batch effect correction, with cells colored by Patient ID.
(C) UMAP visualization of the integrated dataset after batch effect correction, with cells colored by Patient ID.

Supplementary Table 1:
DEGs between PIAS1^{+} and PIAS1^{-} cells in T cells from integrated dataset and their fold change (log2 scale) and adjusted p-value (padj).

Gene	Log2FC	AdjPval

PIAS1	18.38435367	0
RPS29	-0.671580965	2.36215155991722e-35
RPS17	-0.886016528	3.07338265438733e-35
RPL21	-0.618987112	1.8165582677362e-34
RPL31	-0.636590936	1.18356698162442e-32
RPL13A	-0.593121068	1.66310936706743e-32
RPL7	-0.677299164	1.52139403221988e-31
RPL27A	-0.62092069	1.92128275486375e-31
RPS20	-0.66452167	2.43432390317941e-31
RPL9	-0.598954994	4.18873607346243e-28
RPS10	-0.666193288	8.04572610401297e-26
RPL23	-0.581418243	8.34671236590964e-23
TRAC	-0.626336478	8.11977778258968e-21
NDUFA11	-0.917382419	1.91190745691092e-20
PSMA2	-0.983743474	9.99908970449707e-20
ATP5MD	-0.851818145	9.55136126831541e-19
ATP5F1E	-0.642526069	1.5666076066541e-18
RACK1	-0.58099573	4.52733157242686e-18
BLOC1S1	-0.883352303	6.16891541542935e-18
POLR2L	-0.622021885	1.73586741090318e-16
SNRPE	-0.917527205	2.99302789502144e-16
EIF3E	-0.649739971	8.14256781456871e-16
TBCA	-0.71948667	3.56168804461751e-15
C19orf53	-0.602101229	1.14626338784067e-14
ELOB	-0.650755606	2.11540306811399e-14
UCP2	-0.748531577	2.94902247261278e-14
TRBC2	-0.610987942	4.25071873609923e-14
MT-ND4L	0.977228125	5.52810751378367e-14
ENO1	-0.587836434	9.99645255578271e-14
NDUFB1	-0.63713616	1.0638164445237e-13
ATP5MC3	-0.794992195	1.30488598376028e-13
ROMO1	-0.751128747	4.4289414895938e-13
LAMTOR5	-0.647490027	7.89863086822311e-13
TYMP	-0.616913753	1.68875669369255e-12
MINOS1	-0.608816037	1.98378651202332e-12
SSU72	-0.595312455	4.16458503569219e-11
PPP4C	-0.616165751	4.70365351062309e-11
ATP5F1D	-0.631649412	1.45090214675861e-10

RANBP1	-0.631575437	4.67393740711023e-09
COPS9	-0.697151527	4.99918977600427e-09
ATP5F1B	-0.634754743	6.60216025053056e-08
GZMB	-0.678963463	6.83246854900096e-05
RSRP1	0.841254568	0.000111871
TNFAIP3	0.943906385	0.000139775
MT-ND5	0.612594513	0.001251859

Supplementary Table 2:
DEGs between PIAS1$^+$ and PIAS1$^-$ cells in macrophages from integrated dataset and their fold change (log2 scale) and adjusted p-value (padj).

Gene	Log2FC	AdjPval
PIAS1	16.01859466	0
LMNA	-0.90984832	1.73324504931775e-10
SDF2L1	-0.76003878	1.21942942046201e-08
CD59	-0.623948479	7.70389814455045e-08
GTF3C6	-0.612700242	1.33213624530591e-07
RGCC	-0.911728355	3.60470216318571e-06
LIPA	-0.751134117	3.95735983997155e-05
TAP1	-0.603564348	4.86590861813976e-05
HLA-DMB	-0.580019322	8.37850547683654e-05
MMP9	-0.913146976	0.000188817
IDH2	-0.594232158	0.000488105
FCN1	0.623259386	0.001734464
CD9	-0.625824102	0.003426231
CXCL8	-1.394291298	0.013067721
VCAN	0.654114468	0.033427244
PPIF	-0.683205306	0.049047109

Supplementary Table 3:
DEGs between PIAS1$^+$ and PIAS1$^-$ cells fibroblasts from integrated dataset and their fold change (log2 scale) and adjusted p-value (padj).

Gene	Log2FC	AdjPval
PIAS1	15.95492217	0
RPS20	-1.292090989	3.18141650036874e-11
NDUFC2	-1.145226632	3.83473758455641e-10
RPL27A	-1.162979124	4.44145657244097e-10
VKORC1	-1.168491103	5.14426854587401e-08
TAX1BP3	-0.965085683	7.03175073045863e-08
RPL27	-0.700925845	8.05777486737874e-08
RPL37A	-0.689381782	9.9177607977662e-08

NDUFB8	-0.974953962	1.02677581923626e-07
RPS2	-0.714945332	1.04752688177684e-07
CRABP2	-1.819877893	2.07798128429104e-07
RPL13A	-0.925981865	1.04209142911798e-06
EIF4A1	-0.7827762	2.96756132511171e-06
RPL23	-0.789427447	4.80247955689512e-06
CNPY2	-1.032648675	1.61664310773771e-05
GABARAP	0.581494379	1.95597620140447e-05
RPL10A	-0.606632967	2.1274782885542e-05
RPL21	-0.808707306	2.40924427316035e-05
BLOC1S1	-0.740005834	2.42355480450001e-05
SNRPG	-0.604217063	3.5957974370167e-05
AP000769.1	0.749678851	4.76260977566312e-05
IGFBP6	-0.924153061	8.50810897426539e-05
TNFRSF12A	-0.760971072	9.50985842588824e-05
C6orf48	-0.934065659	9.81188418182646e-05
RPS17	-1.218737558	0.000117095
NDUFA11	-0.772156774	0.000121625
RPS29	-0.72279811	0.000140854
SEC23A	-0.855513744	0.000163443
TMSB4X	-0.615450979	0.000209291
SUMO3	-0.843057902	0.000265791
DAPK3	-0.85339823	0.000416545
LOXL1	-0.883112844	0.000518976
ARL2	-0.649200637	0.000650021
RPSA	-0.591498311	0.001352275
MT1E	-0.653634865	0.00139964
PDLIM4	-0.644070597	0.001608275
HSD17B10	-0.624461508	0.002311716
S100A16	-0.624127701	0.002621898
SLC16A3	-0.88518851	0.002703767
PDLIM2	-0.841639324	0.003540612
SNRPE	-0.592557892	0.003961095
CARHSP1	-0.60009268	0.005832881
MRPS15	-0.847880152	0.007027168
PSMA1	-0.595455717	0.009599618
YIF1A	-0.603466066	0.010314534
PLP2	-0.639136126	0.011419553

MRPL41	-0.616038217	0.014973972
CEBPB	-0.662169494	0.019082985
MGAT1	-0.704240968	0.021653665
TMEM14B	-0.688101908	0.023730623
ERP44	-0.687943142	0.026258661
GAS1	-0.650038652	0.026284022
RNF145	-0.632653294	0.033140549
